# First Molecular-Based Confirmation of *Dermacentor marginatus* and Associated *Rickettsia raoultii* and *Anaplasma marginale* in the Hindu Kush Mountain Range

**DOI:** 10.3390/ani13233686

**Published:** 2023-11-28

**Authors:** Iftikhar Ahmad, Shafi Ullah, Abdulaziz Alouffi, Mashal M. Almutairi, Muhammad Numan, Tetsuya Tanaka, Shun-Chung Chang, Chien-Chin Chen, Abid Ali

**Affiliations:** 1 Department of Zoology, Abdul Wali Khan University Mardan, Mardan 23200, Pakistanshafi_ullah@awkum.edu.pk (S.U.);; 2 King Abdulaziz City for Science and Technology, Riyadh 12354, Saudi Arabia; asn1950r@gmail.com; 3 Department of Pharmacology and Toxicology, College of Pharmacy, King Saud University, Riyadh 11451, Saudi Arabia; 4 Laboratory of Infectious Diseases, Joint Faculty of Veterinary Medicine, Kagoshima University, Kagoshima 890-0065, Japan; 5 Department of Emergency Medicine, Ditmanson Medical Foundation Chia-Yi Christian Hospital, Chiayi 60002, Taiwan; 6 Department of Pathology, Ditmanson Medical Foundation Chia-Yi Christian Hospital, Chiayi 60002, Taiwan; hlmarkc@gmail.com; 7 Department of Cosmetic Science, Chia Nan University of Pharmacy and Science, Tainan 717, Taiwan; 8 Ph.D. Program in Translational Medicine, Rong Hsing Research Center for Translational Medicine, National Chung Hsing University, Taichung 402, Taiwan; 9 Department of Biotechnology and Bioindustry Sciences, College of Bioscience and Biotechnology, National Cheng Kung University, Tainan 701, Taiwan

**Keywords:** *Dermacentor marginatus*, *Anaplasma marginale*, *Rickettsia raoultii*, phylogeny, Pakistan

## Abstract

**Simple Summary:**

*Dermacentor* ticks have a wide geographic range with an uneven distribution in the globe. They are not scientifically well known because they survive in hard topographic and harsh climatic regions along with elevated mountains. Many mammals serve as a primary host for *Dermacentor* ticks, like many other tick species. The present study aimed to provide the first morphological and molecular confirmation of *Dermacentor marginatus* and its related pathogens like *Anaplasma marginale* and *Rickettsia raoultii* in Pakistan. In this study, a total of 26 specimens (19 males and 7 females) were collected from goats and morphologically identified. A subset of 18 specimens were subjected for the molecular characterization of ticks and associated pathogen detection. In the BLAST and phylogenetic analyses, *D. marginatus* and their associated pathogen sequences showed close resemblance with their corresponding species. In the present study, we reported the first genetic characterization of *D. marginatus* and associated *A. marginale* and *R. raoultii* in Pakistan. Due to the difficult access and harsh climate, it is important to investigate the ticks and related pathogens in the northern parts of Pakistan due to their zoonotic threats.

**Abstract:**

Ticks of the genus *Dermacentor* Koch, 1844 (Acari: Ixodidae) are poorly known systematically due to their habitation in harsh topographic environments and high mountains. *Dermacentor* ticks are diversely distributed in the Palearctic, Nearctic, and Oriental regions. There is no available information on the occurrence of *Dermacentor marginatus* in Pakistan; thus, the current investigation aimed the first morphological and molecular confirmation of this species and associated *Anaplasma marginale* and *Rickettsia raoultii*. Ticks were collected from goats (*Capra hircus*) and morphologically identified. Genomic DNA was extracted from 18/26 (69.23%) tick specimens, including 11 males and 7 females (1 unfed and 6 fed females). Extracted DNA was subjected to PCR for the amplification of genetic markers like 16S rDNA and *cox1* for ticks, 16S rDNA for *Anaplasma* spp., and *gltA* and *ompB* for *Rickettsia* spp. A total of 26 *D. marginatus* ticks composed of 19 males (73.07%) and 7 females (26.9%) [1 (3.84%) unfed and 6 (23.07%) fed females] were collected from goats. According to amplicons via BLAST analysis, the 16S rDNA sequence showed 97.28–98.85% identity and the *cox1* sequence showed 95.82–98.03% identity with *D. marginatus.* Additionally, the 16S rDNA sequence for *Anaplasma* sp. was detected in *D. marginatus* that showed 100% identity with *Anaplasma marginale*. Rickettsial *gltA* and *ompB* sequences for *Rickettsia* sp. showed 100% identity with *Rickettsia raoultii*. In phylogenetic analysis, ticks’ 16S rDNA and *cox1* sequences clustered with the same species. In phylogenetic analysis, *A. marginale* based on 16 rDNA clustered with *A. marginale*, while *gltA* and *ompB* sequences clustered with *R. raoultii*. This is the first study on the genetic characterization of *D. marginatus* and associated *A. marginale* and *R. raoultii* in Pakistan. The northern areas of Pakistan, which need to be explored in terms of ticks and associated pathogens due to their zoonotic threats, have been neglected due to the inaccessible climatic conditions.

## 1. Introduction

*Dermacentor* Koch, 1844 (Acari: Ixodidae) ticks have a wide geographical range with an uneven distribution, and not a single species is reported from poles and remote islands. Like many other tick species, mammals act as a common host for *Dermacentor* ticks [1,2]. The adult ticks mainly feed on medium- to large-sized mammals, while the larvae feed on small mammals [3]. *Dermacentor* species are important from veterinary and public health perspectives, parasitizing artiodactyl, carnivores, rodents, and insectivores [4] and transmitting different protozoans and bacteria like *Rickettsia* spp. and *Anaplasma* spp. [1,5]. The greatest diversity of these ticks is confined to the Palearctic, Nearctic, and Oriental regions, while only a single species *Dermacentor steini* has been reported from the Australasian zoogeographical region [6].

Difficulties persist in the morphological separation of *Dermacentor* ticks in species complex [7,8]. Recently, *Dermacentor atrosignatus* has been reinstated as *Dermacentor tricuspis*, and *D. atrosignatus* has been considered as a synonym of *D. tricuspis* [9,10]. A single species of the genus *Dermacentor*, i.e., *Dermacentor raskemensis*, has been confirmed and reported from Pakistan based on the collection of immature and adult stages [11,12,13].

*Dermacentor marginatus* was named by German vulgars as ‘Schafzecke’ (sheep tick) due to its infestation with domestic sheep, but it has been also recorded from domestic and wild hosts including cattle, goat, dog, horse, hedgehog, hare, deer, wild boar, and hare [14,15]. This tick generally inhabits steppes, alpine steppes, forest steppes, and semi-desert steppes, particularly open sheep meadows [16]. *D. marginatus* is an ornate tick, geographically distributed from southern Europe to China [15]. The life cycle takes 1–2 years, and its developmental stages are interrupted by low temperatures or by snow cover in winter [17]. Previously, Pakistan was considered out of the adaptation range for *D. marginatus* [6].

These species are important vectors of microorganisms that cause diseases in domestic and wild animals and humans [3]. *D. marginatus* ticks act as potential vectors for the rickettsial group causing zoonotic diseases, which has an important role in the eco-epidemiology of rickettsiosis [18]. The *Rickettsia* species are divided on the basis of genotype and phenotypic characteristics into four major groups, namely spotted fever, typus, bellii, and limoniae groups [19]. *Rickettsia* species have been reported from *D. marginatus*, which include *Rickettsia felis*, *Rickettsia monacensis*, *Rickettsia raoultii*, *Rickettsia slovaca*, and “*Candidatus* Rickettsia rioja” [20,21,22].

There are insufficient data available on *Dermacentor* ticks and their associated pathogens in Pakistan. To the best of our knowledge, this is the first study to report *D. marginatus* and their associated pathogens by using genetic markers to spot the occurrence of this tick in the Hindu Kush Mountain Range in the northern areas of Khyber Pakhtunkhwa (KP), Pakistan. 

## 2. Materials and Methods

### 2.1. Ethical Statement 

For the current study, ethical approval was obtained from the Advanced Studies and Research Board of the Faculty of Chemical and Life Sciences, with notification number Dir/A&R/AWKUM/2022/9674, Abdul Wali Khan University Mardan, KP, Pakistan. Oral permissions were taken from the animal owners.

### 2.2. Study Area 

**Figure 1:**
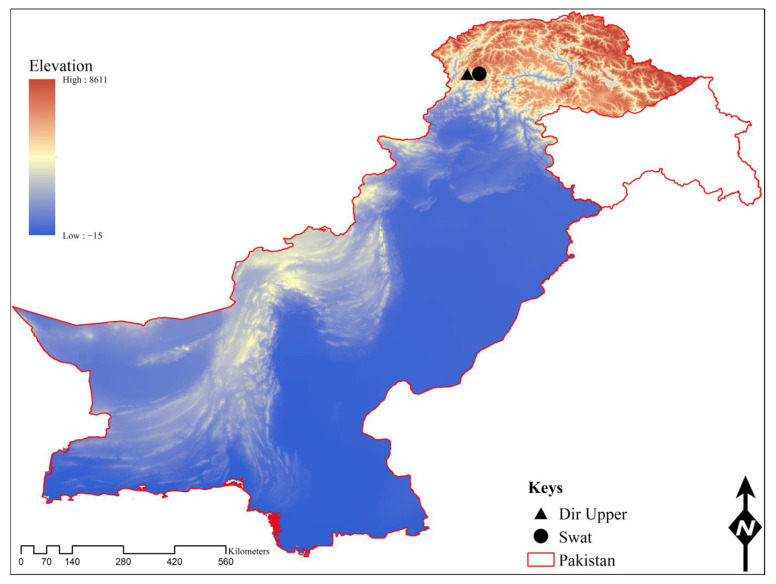
Tick collection site in the border region (Badgoi pass) of districts Dir Upper and Swat of the Hindu Kush Mountain Range.

This study was conducted in the border region of the following districts: Dir Upper (35°30′00.1″ N, 72°20′43.0″ E) and Swat (35°34′27.3″ N 72°22′07.7″ E)—Badgoi pass of the Hindu Kush Mountain Range, the northern part of the KP province of Pakistan. The Badgoi pass is the link between districts Dir Upper and Swat, with an altitude of nearly 3520 m above sea level. The physiography of the area is composed of gently steep, rocky mountains covered with snow in winter and turned into green pastures in summer. The summer season is from moderate to warm, and temperatures rapidly fall in November to February. The average temperature ranges from −7 °C to 22 °C (climate-data.org). The geographical coordinates of the collection sites were obtained by using a Global Positioning System (GPS), and the map was designed via ArcGIS V. 10.3.1 (Figure 1).

### 2.3. Ticks Collection and Morphological Identification

Tick specimens were collected in June 2022 from goats (*Capra hircus*) in the study area. Ticks were washed with distilled water followed by 70% ethanol to remove the contaminants and extra tissues from the body surface. Ticks were morphologically identified under a stereo-microscope (StereoBlue-euromex SB.1302-1, Arnhem, The Netherlands), and their morphological characters were compared with the standard identification keys [23,24].

### 2.4. DNA Extraction and PCR

A total of 18/26 (69.23%) *Dermacentor* specimens comprising 9 specimens from each locations: Dir Upper (6 males and 1 unfed and 2 fed females) and Swat (5 males and 4 fed females) were subjected to genomic DNA extraction. The specimens were washed and crushed in a sterilized 1.5 mL Eppendorf tube. The genomic DNA was extracted by the phenol-chloroform method [25]. The DNA pellet was hydrated by adding 30 µL “nuclease-free” PCR water and quantified with NanoDrop (Optizen, Daejeon, South Korea).

A conventional PCR (GE-96G, BIOER, Hangzhou, China) was performed to amplify the16S rRNA and *cox1* genes for tick identification (Table 1). All genomic DNA samples were utilized to test for associated pathogens through the amplification of the 16S rRNA partial gene for *Anaplasma* species and the *gltA* and *ompB* partial genes for *Rickettsia* species (Table 1).

Each PCR mixture was prepared in 25 µL, composed of 2 µL genomic DNA (100 ng/µL), 1 µL each (forward and reverse) primer (10 µM), 8.5 µL PCR water “nuclease-free”, and 12.5 µL Dream*Taq* green MasterMix (2×) (Thermo Fisher Scientific, Inc., Waltham, MA, USA). Each of the PCRs contained a positive control (DNA of *Rickettsia massiliae* and *Anaplasma marginale* for pathogens, and DNA of *Rhipicephalus microplus* for ticks) as well as a negative control (PCR water that was “nuclease-free” rather than DNA). The PCR products were run on a 2% agarose gel electrophoresis and observed via Gel documentation system (BioDoc-It™ Imaging Systems, UVP, LLC., Upland, CA, USA).

### 2.5. DNA Sequencing and Phylogenetic Analysis

The amplicons were purified by using a Gene Clean II Kit (Qbiogene, Il-lkirch, France) as per the manufacturer’s protocol. The PCR-amplified products were sent for bidirectional sequencing to Macrogen, Inc. (Seoul, Republic of Korea). The obtained sequences were trimmed to remove the contaminated and poor reading regions via SeqMan v. 5 (DNASTAR, Inc., Madison, WI, USA). The trimmed sequences were subjected to the Basic Local Alignment Search Tool (BLAST) in the National Center for Biotechnology Information (NCBI). The high-percentage identity sequences were downloaded in FASTA format from the NCBI for phylogenetic analyses. The downloaded sequences were aligned with the obtained sequences and an outgroup using ClustalW multiple alignments [31] in BioEdit Sequence Alignment Editor v. 7.0.5 [32]. The coding sequences were aligned by using MUSCLE statistical algorithms [33]. The phylogenetic trees were constructed through the maximum likelihood method and the Tamura–Nei model with a 1000 bootstrapping value in MEGA-X [34].

## 3. Results

### 3.1. Tick Record

Goat herds were observed for tick collection in 11 different localities in Badgoi pass Dir Upper and Swat. Among these goat herds, 11 goats (5 males and 6 females) were infested by *D. marginatus* ticks, counting 26 (19 males and 1 unfed and 6 fed females) ticks. Among these, 16 ticks (13 males and 1 unfed and 2 fed females) were from Dir Upper and 10 ticks (6 males and 4 fed females) from Swat. The *Dermacentor*-infested goats were of three age groups, namely 4 adults, 4 young, and 3 kids. Besides *D. marginatus* ticks, these goats were co-infested by *Haemaphysalis montgomeryi*. A total of 45 *H. montgomeryi* ticks including 23 males and 22 females (6 unfed and 16 fed females) were collected. Environmental and topographic conditions as well as the host records are mentioned in Table 2.

### 3.2. D. marginatus Male

**Figure 2:**
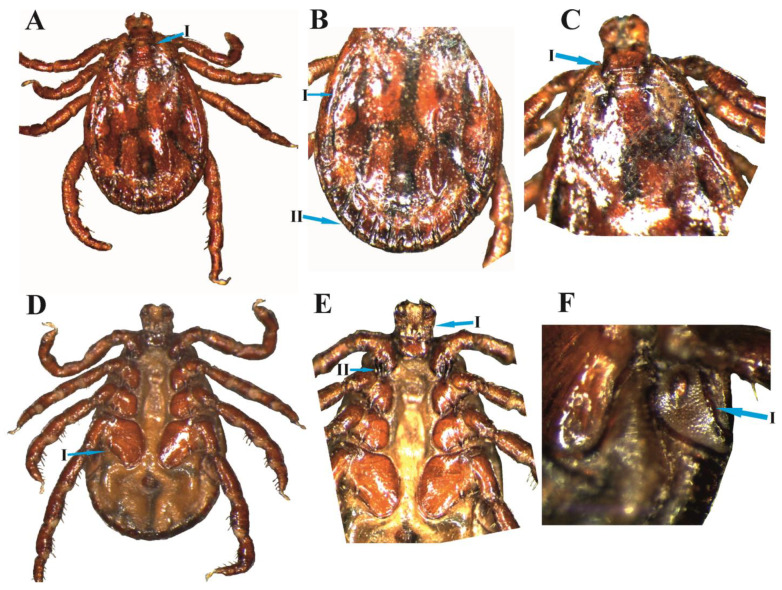
*D. marginatus* male: (**A**) male dorsum (I: cervical groove), (**B**) posterior dorsum (I: lateral groove, II: festoons), (**C**) anterior dorsum (I: basis capitulum), (**D**) male venter (I: coxa IV spur), (**E**); anterior venter (I: capitulum ventrally, II: first coxa spurs), and (**F**); posterior venter (I: spiracle plate).

Idiosoma: The body is medium-sized with an elongated oval shape. Conscutum is inornate, dark reddish with irregular black patches. Cervical grooves are short, deep, and comma-shaped converging anteriorly and posteriorly. Punctuations are deep, not uniform, and densely distributed in the anterior (Figure 2A(I)). The marginal groove does not enclose the posterior festoon and starts from the last festoon and ends at the point of 2nd coxae (Figure 2B(I, II)). The width of the basis capitulum is greater than its length with moderately pointed broad cornua (Figure 2C(I)). Legs: dorsal side segments are with pale yellow color spots. Coxa I is small with paired spurs equal in size (Figure 2E(II)). A single external spur is present on coxae II, III, and IV. Coxa IV is large and broader anteriorly while becoming narrow posteriorly (Figure 2D(I)). The coxa size progressively increases from coxae I to IV. Hypostome is club-shaped accompanied by 3/3 dentation (Figure 2E(I)). Spiracular plates are broad at the upper side and gradually pointed posteriorly. Goblet cells are around the macula. Macula is parallel with the lateral margin (Figure 2F(I)).

### 3.3. D. marginatus Female

**Figure 3:**
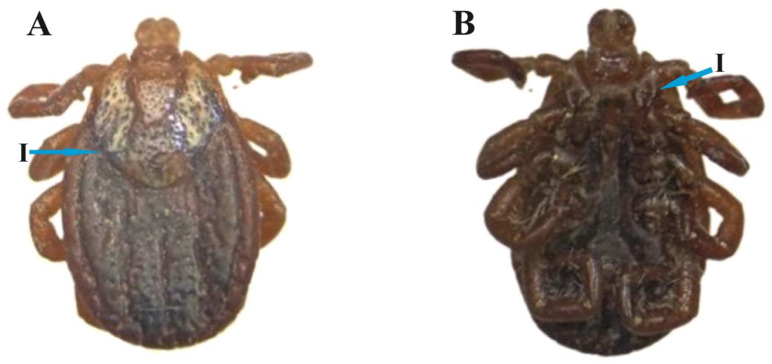
*D. marginatus*: (**A**) female dorsum (I: scutum shape) and (**B**) female venter (I: first coxa spurs).

Body idiosoma: The body is dark reddish and oval-shaped. The scutum is moderate-sized and sub-ovate in shape. The scutum posteriorly diverges at mid-position and gradually converges to a narrow-rounded position (Figure 3A(I)). The cervical groove is deep and curved inside. The alloscutum has inner deep grooves, with posterior–marginal depressions. Capitulum width is greater than length. The basis capitulum is broader as compared to the length. Punctuations are deep, not uniform, and irregularly distributed (Figure 3A). Coxa I is small with a paired spur (Figure 3B(I)).

### 3.4. Molecular and Phylogenetic Analysis of D. marginatus

**Figure 4:**
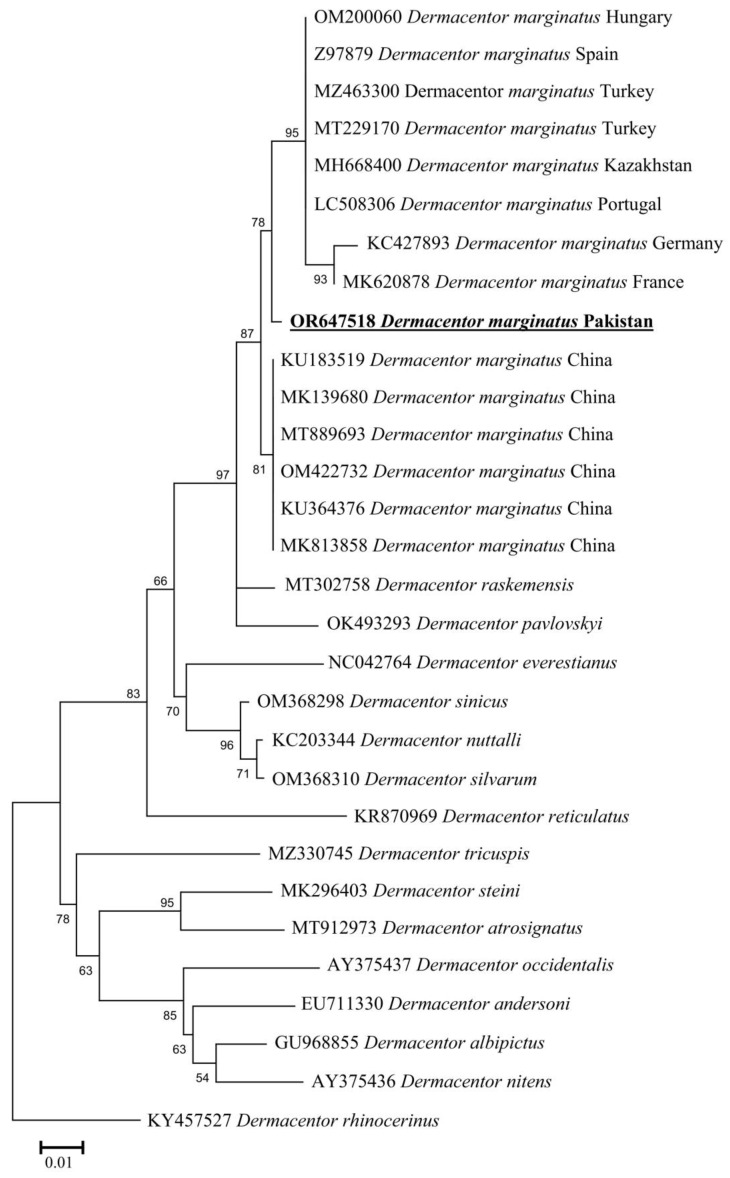
Maximum-likelihood phylogenetic analysis of nucleotide sequences of 16S rDNA partial sequences for *Dermacentor* spp. The sequences are represented by their GenBank accession number followed by the name of species and countries (when applicable). The obtained sequence in the present study is indicated in bold and underlined (accession number: OR647518).

**Figure 5:**
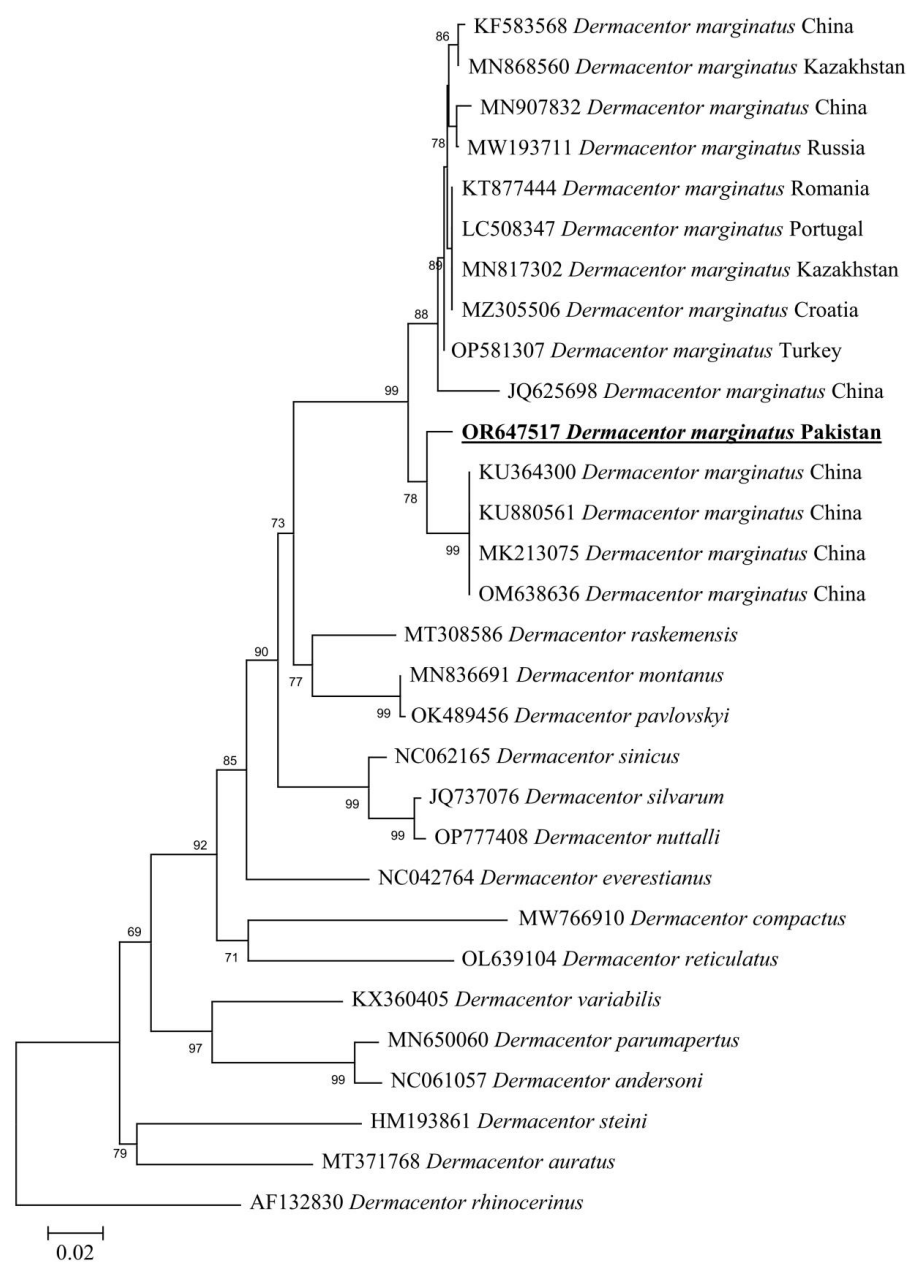
Maximum-likelihood phylogenetic analysis of nucleotide sequences of *cox1* partial sequences for *Dermacentor* spp. The sequences are represented by their GenBank accession number followed by the name of species and countries (when applicable). The obtained sequence in the present study is indicated in bold and underlined (accession number: OR647517).

By the BLAST results, the 16S rDNA partial sequence showed a 97.28–98.85% maximum identity range with *D. marginatus* reported from various countries: China (98.85%—OM422732, 98.77%—KU183519, 98.61%—MK139680, 98.51%—KU364376, 98.34%—MT889693, and 98.13%—MK813858), Kazakhstan (97.93%—MH668400), Turkey (97.84%—MT229170), Portugal (97.82%—LC508306), Germany (96.79%—KC427893), Hungary (97.78%—OM200060), Turkey (97.74%—MZ463300), Spain (97.71%—Z97879), and France (97.28%—MK620878). On the other hand, the *cox1* sequence showed a 95.82–98.03% maximum identity range with *D. marginatus* reported from different countries: Turkey (98.03%—OP581307), Croatia (97.87%—MZ305506), China (97.87%—KU364300, 97.86%—MK213075, 97.69%—KU880561, 97.65%—KF583568, 97.65%—OM638636, 97.51%—MN907832, and 95.82%—JQ625698), Kazakhstan (97.85%—MN817302 and 97.79%—MN868560), Portugal (97.70%—LC508347), Romania (97.61%—KT877444), and Russia (97.37%—MW193711). In the phylogenetic analysis, these 16S rDNA and *cox1* sequences were clustered with *D. marginatus* reported from the Palearctic region (Figure 4 and Figure 5).

### 3.5. Molecular Screening and Phylogenetic Analysis of D. marginatus Associated Pathogens

**Figure 6:**
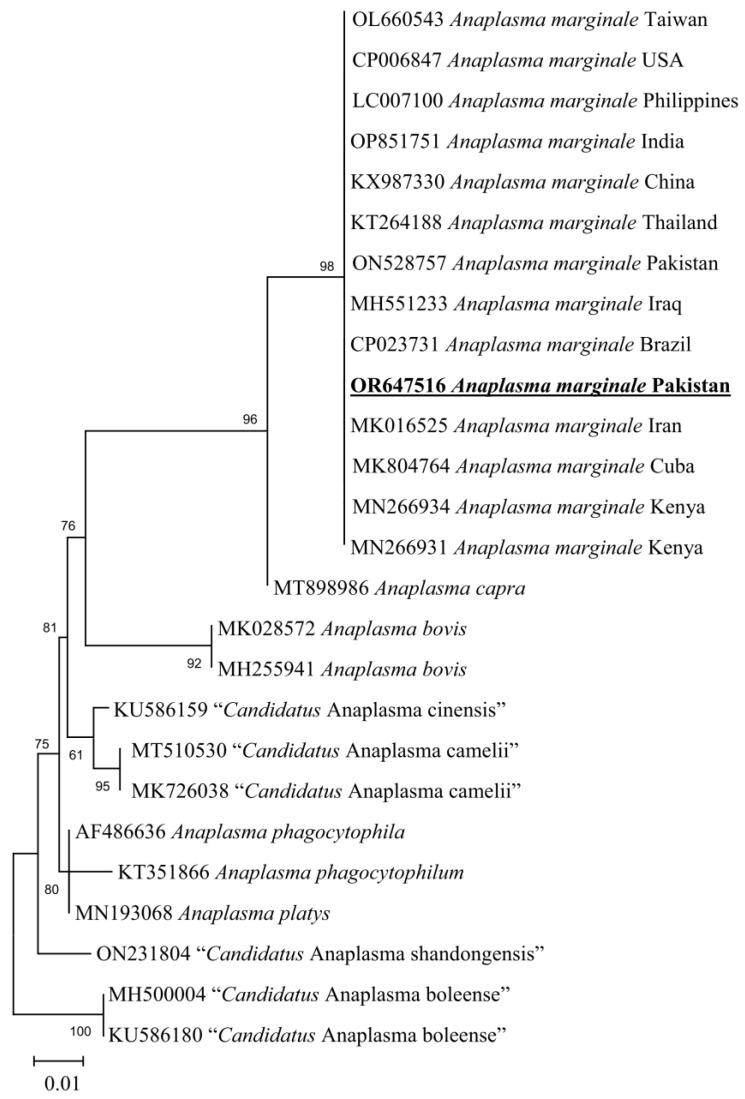
Maximum-likelihood phylogenetic analysis of nucleotide sequences of 16S rDNA partial sequences for *Anaplasma* spp. The sequences are represented by their GenBank accession number followed by the name of species and countries (when applicable). The obtained sequence in the present study is indicated in bold and underlined (accession number: OR647516).

*Anaplasma marginale* (2/18; 11.11%) and *R. raoultii* (2/18; 11.11%) were documented in *D. marginatus* ticks. The 16S rDNA sequence for *Anaplasma* sp. showed 100% identity with *Anaplasma marginale* and phylogenetically clustered with the same species reported from Pakistan (ON528757), Taiwan (OL660543), USA (CP006847), Philippines (LC007100), India (OP851751), China (KX987330), Thailand (KT264188), Iraq (MH551233), Brazil (CP023731), Iran (MK016525), Cuba (MK804764), and Kenya (MN266931 and MN266934) (Figure 6).

**Figure 7:**
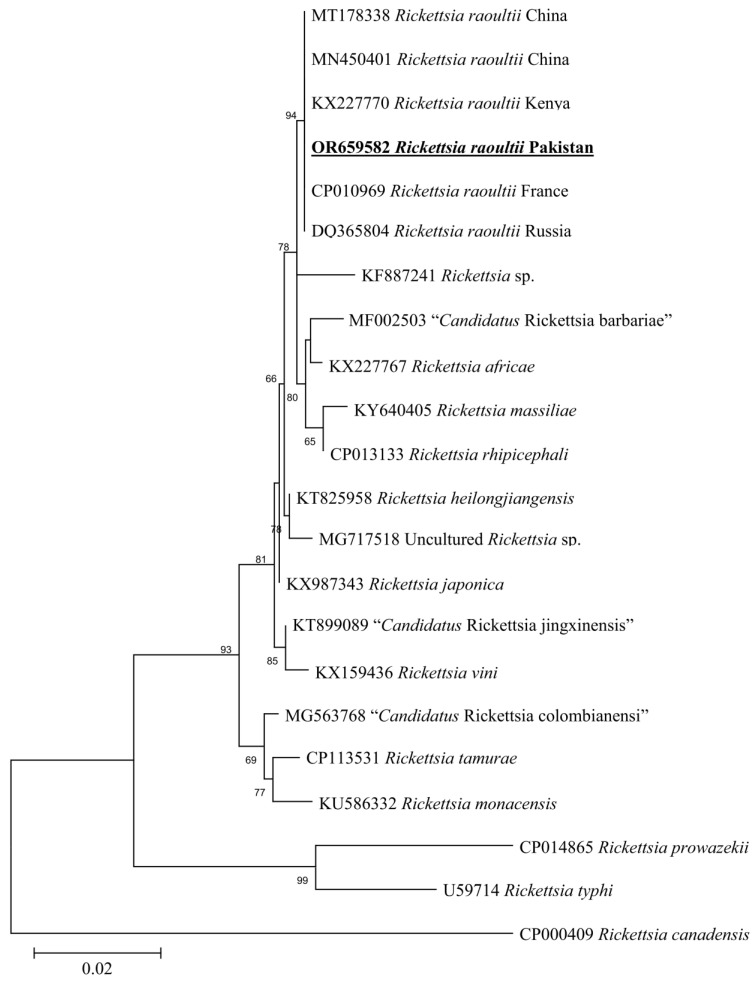
Maximum-likelihood phylogenetic analysis of nucleotide sequences of *gltA* partial sequences for *Rickettsia* spp. The sequences are represented by their GenBank accession number followed by the name of species and countries (when applicable). The obtained sequence in the present study is indicated in bold and underlined (accession number: OR659582).

**Figure 8:**
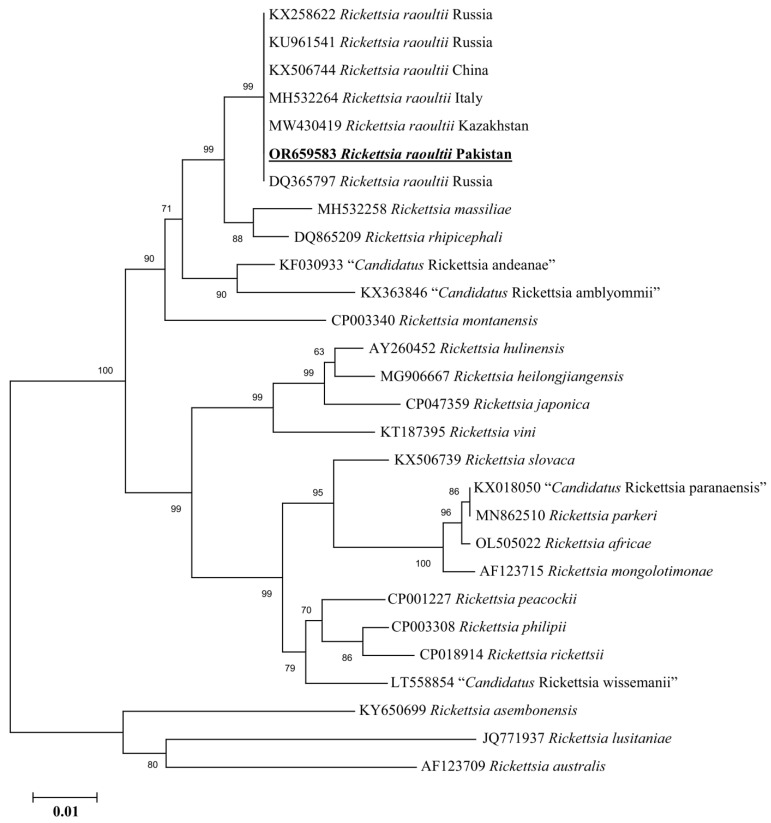
Maximum-likelihood phylogenetic analysis of nucleotide sequences of *ompB* partial sequences for *Rickettsia* spp. The sequences are represented by their GenBank accession number followed by the name of species and countries (when applicable). The obtained sequence in the present study is indicated in bold and underlined (accession number: OR659583).

The rickettsial *gltA* sequence showed 100% identity with the *Rickettsia raoultii* reported from China (MT178338 and MN450401), Kenya (KX227770), France (CP010969), and Russia (DQ365804). Meanwhile, the rickettsial *ompB* sequence showed 100% identity with the *Rickettsia raoultii* from Russia (KX258622), Russia (KU961541), China (KX506744), Italy (MH532264), Kazakhstan (MW430419), and Russia (DQ365797). In the phylogenetic trees, these rickettsial *gltA* and *ompB* sequences were clustered with the corresponding species (Figure 7 and Figure 8).

## 4. Discussion

The genus *Dermacentor* is composed of 44 tick species, originated in Africa and spread to the New World through Palearctic zoogeographical regions [2,6,35]. One of the most diverse groups with fewer number of species [35], the systematic knowledge and evolutionary history of this genus is still poorly known [1,35,36]. Herein, ticks were collected from goats in the Hindu Kush Mountain Range at Badgoi pass and identified morphologically and phylogenetically as *D. marginatus*. This is the first confirmed host record and the preliminary phylogenetic position of *D. marginatus* from Pakistan. Despite being widely distributed throughout the Palearctic region, the *D. marginatus* tick has not yet been identified in Pakistan [6,24]. This raises concerns about *D. marginatus* whether it is considered an exotic or native species in the country. Environmentalists and public health professionals face an increasing concern about the spread of exotic ticks into novel ecosystems [37].

*D. marginatus* ticks are difficult to morphologically identify, as *Dermacentor nuttalli, Dermacentor ushakovae, Dermacentor niveus*, and *Dermacentor silvarum* were considered as synonyms; in this case, [6] these species were confirmed as *D. marginatus* species complex. The morphological description of the male and female *D. marginatus* has revealed that both sexes have white enamel on the scutum and conscutum [4]. The collected male tick during this study has reddish-black pigmentation and with no ornamentation on the conscutum; this phenomenon has been previously reported in *Dermacentor* ticks, i.e., *Dermacentor albipictus* [38].

Together with morphological identification, the genetic characterization of ticks has been considered as an important component in understanding tick systematic and evolutionary history [39]. In this study, we employed DNA barcoding for the precise identification of *D. marginatus*. The 16S rRNA and *cox1* genes are important in marking tick phylogenies due to the availability of the dataset in GenBank for these sequences as well as containing hypervariable regions [40]. In the phylogenetic trees, the obtained 16S rDNA and *cox1* sequences found a close resemblance with the sequences of *D. marginatus* reported from different countries.

Exponential rates of ticks and tick-borne diseases due to climate change have threatened global health. In Pakistan, serious attention to regular surveillance of ticks and tick-borne pathogens has not been paid yet. However, previous studies have highlighted various ticks as zoonotic risks regarding the detection of pathogens like “*Candidatus* Rickettsia shennongii”, *Rickettsia massiliae*, *Rickettsia aeschlimannii*, *Rickettsia conorii*, *Rickettsia hoogstraalii*, *Coxiella burnetii*, *Borrelia* sp., *A. marginale*, and *Theileria annulata* [41,42,43,44,45]. Although the occurrence of *D. marginatus* in Pakistan and the detection of *A. marginale* and *R. raoultii* in this tick are a serious concern, as previously mentioned, this species has been found to be involved in the transmission of various pathogens like *Rickettsia slovaca*, *A. marginale Coxiella* spp., *Rickettsia* spp., *Shigella* spp., and *Francisella* spp. [46]. To assist the public and veterinary health surveillance, the current study monitored the abundance and distribution of the utmost important goat-associated *Dermacentor* ticks in the highlands of the northern region of Pakistan. Moreover, the molecular detection of pathogens like *R. raoultii* and *A. marginale* highlights the zoonotic risks due to these parasites. Future research must address the gaps regarding the presence of *D. marginatus* in various topographic regions of Pakistan in order to understand whether this species is established or is invasive.

## 5. Conclusions

This study presents a comprehensive report on *D. marginatus* ticks and associated *A. marginale* and *R. raoultii* for the first time in Pakistan. Molecular identification and host record confirmed that the ticks of the genus *Dermacentor* mostly prefer cold climatic conditions. Furthermore, pathogens were detected that have a zoonotic threat to the livestock-holders of the study areas. Moreover, this study may assist in understanding the epidemiology and systematic history of *Dermacentor* species.

## Figures and Tables

**Table 1:** List of primers utilized for the amplification of target DNA sequences.

Samples	Target Genes	Sequences (5′-3′)	Size bp	PCR Conditions	References
Ticks	16S rRNA	16S+1-CCGGTCTGAACTCAGATCAAGT 16S−1-CTCAATGATTTTTTAAATTGCTG	460	95 °C 3 min, 40 × (95 °C 30 s, 55 °C 60 s, 72 °C 1 min), 72 °C 7 min	[26]
	*cox1*	HC02198-TAAACTTCAGGGTGACCAAAAAATCA LCO1490-GGTCAACAAATCATAAAGATATTGG	712	95 °C 30 s, 40 × (95 °C 30 s, 48 °C 30 s, 72 °C 1 min), 72 °C 5 min	[27]
Pathogens	16S rRNA	EHR16SD-GGTACCYACAGAAGAAGTCC EHR16SR-TAGCACTCATCGTTTACAGC	344	95 °C 5 min, 35 × (95 °C 30 s, 55 °C 30 s, 72 °C 90 s), 72 °C 5 min	[28]
	*gltA*	CS-78 GCAAGTATCGGTGAGGATGTAAT CS-323 GCTTCCTTAAAATTCAATAAATCAGGAT	401	95 °C 3 min, 40 × (95 °C 15 s, 48 °C 30 s, 72 °C 30 s) 72 °C 7 min	[29]
	*ompB*	120-M59-CCGCAGGGTTGGTAACTGC 120-807-CCTTTTAGATTACCGCCTAA	862	95 °C 3 min, 40 × (95 °C 30 s, 50 °C 30 s, 68 °C 1 min 30 s), 68 °C 7 min	[30]

**Table 2:** Table describing the comprehensive data regarding the collection of *D. marginatus* in Hindu Kush Mountain Range.

District	Host	Sex	Age Group	Temperature and Humidity	Place of Collection and Elevation	Tick Species	
						Targeted Species: *D. marginatus*	Accompanied Species: *H. montgomeryi*
Dir Upper	Goat	Female	Adult (above 1 year)	12 °C, 78%	Kund Banda, 3347 m	2M	2M, 1F *
Dir Upper	Goat	Male	Young (above 6 months–1 year)	13 °C, 78%	Gaedar Banda, 3344 m	1M, 2F	3M
Dir Upper	Goat	Male	Kid (below 6 months)	13 °C, 78%	Bend Banda, 3340 m	2M	4F
Dir Upper	Goat	Female	Young (above 6 months–1 year)	15 °C, 78%	Jan Shahi, 3340 m	2M, 1F *	1M, 3F
Dir Upper	Goat	Female	Adult (above 1 year)	13 °C, 78%	Cherry Banda, 3349 m	2M	2M
Dir Upper	Goat	Female	Kid (below 6 months)	14 °C, 78%	Dand Banda, 3352 m	1M	4M, 1F *
Dir Upper	Goat	Male	Young (above 6 months–1 year)	14 °C, 78%	Otalshai, 3348 m	3M	2M, 4F
Swat	Goat	Male	Kid (below 6 months)	15 °C, 78%	Dirgal, 3340 m	1M, 3F	4M
Swat	Goat	Female	Adult (above 1 year)	15 °C, 78%	Gorgal, 3345 m	2M	5F
Swat	Goat	Female	Young (above 6 months–1 year)	15 °C, 78%	Landai dara, 3336 m	1M, 1F	3M, 1F *
Swat	Goat	Male	Adult (above 1 year)	15 °C, 78%	Dasht e Liala, 3351 m	2M	2M, 3F *
Total	11 goats	5 males, 6 females	4 adults, 4 young, 3 kids			19M, 7F	23M, 22F

M: male, F *: unfed female and F: fed female.

## Data Availability

The datasets to support the conclusions of this article are given within the article.
